# High-Methionine Diet Attenuates Severity of Arthritis and Modulates IGF-I Related Gene Expressions in an Adjuvant Arthritis Rats Model

**DOI:** 10.1155/2016/9280529

**Published:** 2016-09-25

**Authors:** Mingxin Li, Lidong Zhai, Wanfu Wei

**Affiliations:** ^1^Tianjin Hospital, Jiefangnan Road 406, Tianjin 300210, China; ^2^Department of Anatomy and Histology, Basic Medical College, Tianjin Medical University, Tianjin 300070, China

## Abstract

Rheumatoid arthritis, a synthesized form of adjuvant arthritis exhibited throughout many animal species, inhibits liver function and circulation of IGF-I and contributes to the degradation of skeletal muscle mass. One of the primary goals of the present study is determining whether a high-Methionine (high-Met) diet is capable of reducing the adverse effects of arthritis, namely, loss of body mass. Following adjuvant injection, forty arthritic rats were randomly assigned to either a control group with a basal diet or a high-Met group with the same basal diet + 0.5% Methionine. After 14 days all rats were terminated. The high-Met group exhibited an increase in body weight and food intake in comparison with the control group (*P* < 0.05). High-Met diet debilitated arthritis-induced surges in the gastrocnemius in both atrogin-1 and the MuRF1 expressions; however, it was observed to have little to no effect on atrogin-1 and MuRF1 gene expression in soleus. At the same time, high-Met diet rats experienced a rise in IGF-I, with lowering of IGFBP-3 gene expression in the gastrocnemius and the soleus. These data suggest that arthritis severity can be partly attenuated by high-Met diet.

## 1. Introduction

Adjuvant arthritis is a synthesized variant of rheumatoid arthritis which may be induced in rats via Freund's adjuvant intradermal injection. Following a ten-day period from the point of injection, chronic inflammation and polyarthritis become evident, along with a decrease in general body and muscle mass [1]. Inflammatory cachexia is considered to be a multifactorial process, resulting from a surge in inflammatory regulating entities and exhibiting endocrine modifications [2]. With respect to effects within the endocrine system, an increase in glucocorticoid secretion is observed [3] alongside inhibited release of anabolic hormones, namely, growth hormones (GH), testosterone, and insulin-like growth-factor-1 (IGF-I) [4]. As such, decreases in weight during chronic inflammation may occur partially due to insufficient IGF-I levels present within body.

Muscle atrophy in arthritic rats has been linked to upregulation of atrogin-1 and MuRF-1 which are E3 ubiquitin-ligating enzyme genes and belong to the ubiquitin-proteasome proteolytic pathway [5]. Meanwhile, muscle wasting is also linked to the changes of the muscle IGF-IGFBPs system [1]. IGF-I has been analysed as the prevalent positive coordinator that designates levels of muscle mass [6]. The hormone facilitates a surge in generating muscle mass and aids in assessing satellite cells while additionally inhibiting the upregulation of atrogin-1/MuRF1 [7]. Furthermore, injecting IGF-I eliminated arthritis-induced proliferation in both muscle gene expression factors atrogin-1 and IGFBP-3 [8].

Studies have concluded that the signaling pathway of the insulin-like growth-factor-I (IGF-I) [9] facilitates muscle tissue generation. The mechanism is catalyzed by presence of amino acids, in particular, Methionine (Met). A lack of Met is correlated with a lesser muscle mass [10] and further research has delineated supplementing additional Met into a diet to be highly effective in maintaining healthy muscle mass [11].

The assistive nature of high-Met diets in stimulating skeletal muscle development is well established; however, high-Met diet in dysfunctions related to skeletal muscle states is much less clear. It remains to be seen also, given the minimal research at present, whether high-Met diet mediation may assist in muscle degradation as a result of arthritis which modifies local expression of IGF-I interactions with IGFBPs.

The primary goal of this study therefore is to determine whether a high-Met diet could mitigate the adverse effects of adjuvant arthritis. Research has been undertaken into the resultant dietary effects between expressions of atrogenes, IGF-I, IGFBP-3, and IGFBP-5.

## 2. Material and Methods

### 2.1. Animals and Experimental Design

The present research complied with all Laboratory Animal Ethical Commission guidelines of the Tianjin Hospital of China. Forty male rats at five weeks of age were purchased from the Tianjin Laboratory Animal Public Service Center. Arthritic rats were afflicted as such via intradermal injection, administered in the right paw, and suspension rate 4 mg heat-treated (Freud's adjuvant) Mycobacterium butyricum with 0.1 mL paraffin oil, administered in isoflurane anesthesia conditions. 72 hours after this procedure, rats were placed into cages, 2 rats/cage, and cared for under ambient temperature and lighting: 20–22°C and lights switched on between the hours of 7:30 and 19:30.

Water was in free supply for the rats, which were split into two groups following adjuvant injection. The control group was fed a standard rodent diet [12], whereas the variable high-Met diet group received a surplus 0.5% quantity of Methionine. Food allocations per cage were calculated over a 24-hour period through measurement of the differences between the feeding and what remained at the end of the period. Intake was expressed in g per single rat, per 100 g of total weight. All rats were terminated following a 14-day period. The arthritic index of individual rats was delineated by grading each paw with a value between 0 and 4, with the following criteria: 0: no evidence of swelling, erythema, 1: minor erythema/swelling in at least one digit, 2: the entirety of the paw being evidently subject to moderate inflammation, 3: erythema being evident alongside inflammation spreading to the wrists, and 4: ankylosis and inability to maneuver ankle joints due to severe inflammation. Final scores were deduced through a collective total of the derived scores from each rat paw. Researchers expunged trunk blood into deheated tubes, allowed a clotting period and centrifuged with resultant serums kept at −20°C. Livers and gastrocnemius and soleus muscles (on the left side) were extrapolated, then dissected, and stored to freezing temperatures using liquid nitrogen, kept at −80°C.

### 2.2. RNA Extraction and RT-PCR

Gastrocnemius samples of 100 mg were assimilated. RNA was extrapolated through the TRIzol reagent (Invitrogen, USA). DNase I (Invitrogen, USA) was then added to treat the sample, in accordance with provided guidelines. RNA concentration and integrity were solidified through use of an agarose gel electrophoresis. In the case of RT-PCR analysis, 1 *μ*g gastrocnemius RNA was utilised in cDNA creating with both oligo (dT) 20 and Superscript II reverse transcriptase (provided by Invitrogen, USA). In this case, primers were selected through analysis of existing research to delineate the optimal primer [13] with 18S rDNA as a reference gene. Thermal cycles can be delineated thus; a preincubation period of 95°C for a duration of 10 seconds, next, 40 cycles at 95°C, with denaturing spanning for 15 seconds, and a 60°C annealing stage subsequently occurring for a 30-second period, with 72°C extension procedures for 30 seconds, were undertaken. Final analyses were expressed in relation to 18S rDNA gene, whereby the prevalence of 18S rDNA was set to 1, utilizing a cycle limit of 2(ΔΔCT) method.

### 2.3. Western Blot of IGFBP-3 and IGF-I Determination

IGF product concentration was measured through Western blot. A 2 mL product quantity was diluted within a sample buffer, to then be subjected to intense heating temperatures of 90°C for 2 minutes; samples were then placed onto 1% SDS-12.5% polyacrylamide gels and electrophoresed within a nonreducing context. Homogenous values of intestinal mucosa proteins were extrapolated using a polyacrylamide gel prior to relocation to a polyvinylidene difluoride (PVDF) membrane (sourced from Millipore, Bedford, MA, USA). Specimens then underwent incubation alongside primary IGFBP-3 antibodies (Abcam, US) for a duration of 12 hours at 4°C. PVDF membranes were subsequently kept in a warm environment alongside secondary antibodies goat anti-rabbit IgG-HRP (with products purchased from Santa Cruz Biotechnology, CA, USA) for 2 hours at a temperature of 25°C. Western blots were depicted via a superior chemiluminescence detection kit (Amersham, Arlington Heights, IL, USA). Images were procured with an Alpha Imager 2200 software application provided by the Alpha Innotech Corporation (CA, USA). Uniform distributions were observed among *β*-actin reference proteins across the groups. The protein expression value was ascertained to be the densitometry ratio of both IGFBP-3 and *β*-actin.

### 2.4. Statistical Analysis

 Statistical analyses were undertaken via SPSS 25.0 (Chicago, IL, USA). Student's* t*-tests were utilised to ascertain the variation across the groups, with the statistical significance threshold at *P* < 0.05. Observations are delineated here as the means ± mean standard error (SEM).

## 3. Results

**Figure 1:**
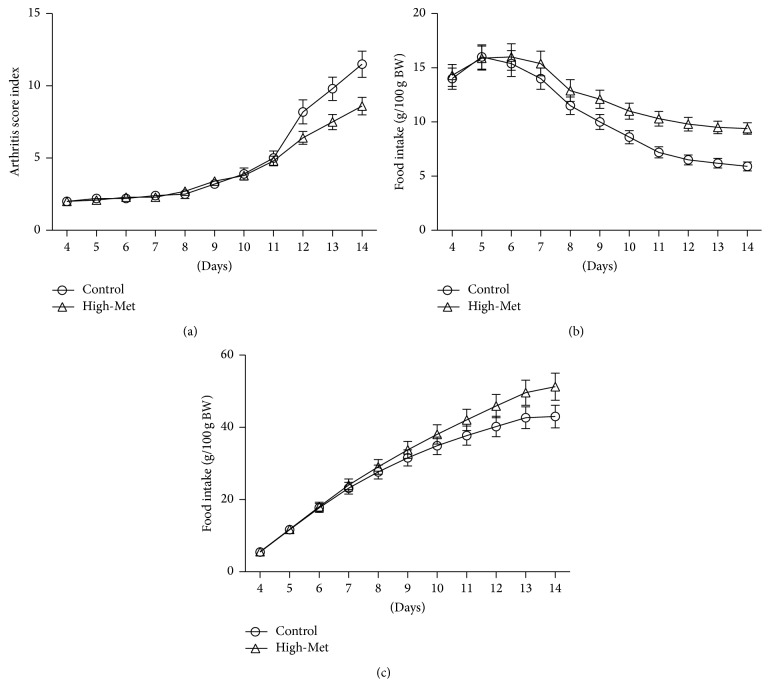
Arthritis index score (a) and consumption of food per 100 g/body weight (b), with the aggregate weight gain development (c) in days 4–14, following adjuvant injections within both the high-Met and control groups. Increased ASI corresponds to decreased food intake. Conversely, high-Met diets led to a rise in food consumption in comparison with the control group. Arthritic rats in the variable group collectively amassed a greater weight gain than the base group. Data are expressed as means ± SEM (*n* = 10).

Arthritis score indices (ASI) developments through measurements of food consumption and weight gain are demonstrated in Figure 1. Throughout the initial stages of illness, prior to day 10 following the adjuvant injection, the arthritic rats developed minor inflammatory reaction within the injected paw. Scores for the arthritic symptoms in the paw ranged between 2 and 4 (Figure 1(a)); however, this was not the case for paws which did not undergo injection. By the tenth day, the poly-articular inflammation began, and by day 14, for the control group, ASI reached the maximal value.

Arthritic symptoms were correlated with a reduction in food consumption (*P* < 0.05) for the 14-day duration (Figure 1(b)). Across both groups, food consumption was lowest between days 13 and 14, which correlated with a steep incline in the ASI. High-Met diet-fed arthritic rats increased their food consumption (Figure 1(b)). In accordance with the hypotheses, control group weight gain was significantly lower than the high-Met diet group, from the ninth day (*P* < 0.05, Figure 1(c)).

**Figure 2:**
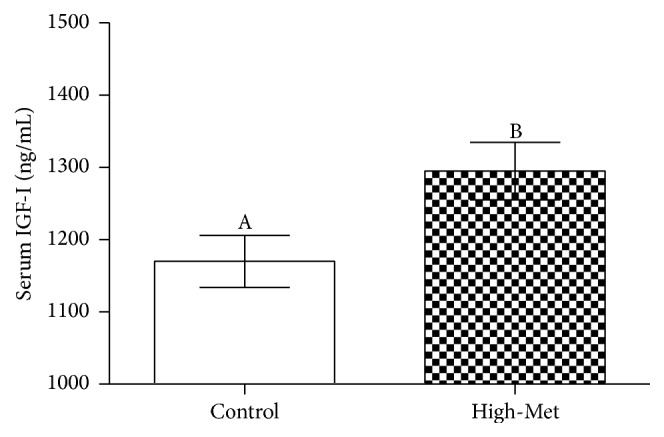
Serum levels of IGF-I when arthritic rats ate a high-Met diet. Data are shown here as means ± SEM (*n* = 8).  ^A,B^The varying letters indicate statistical significance in the divergences of the high-Met and control groups (*P* < 0.05).

**Figure 3:**
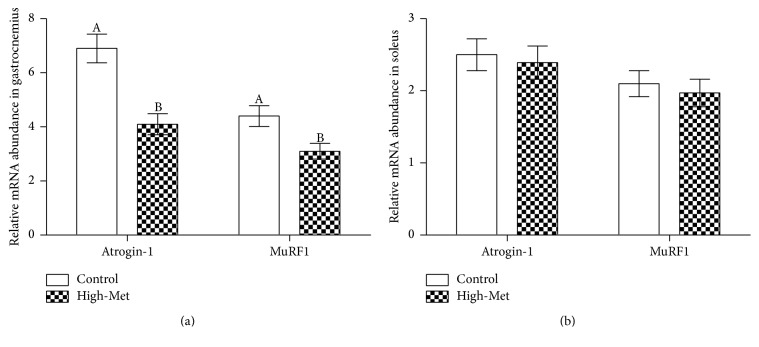
Results of high-Met diet upon the level of mRNA in gastrocnemius (a) and soleus (b). Data is presented as means ± SEM (*n* = 8).  ^A,B^The varying characters indicate the statistically significant differences between the high-Met and control groups (*P* < 0.05).

**Figure 4:**
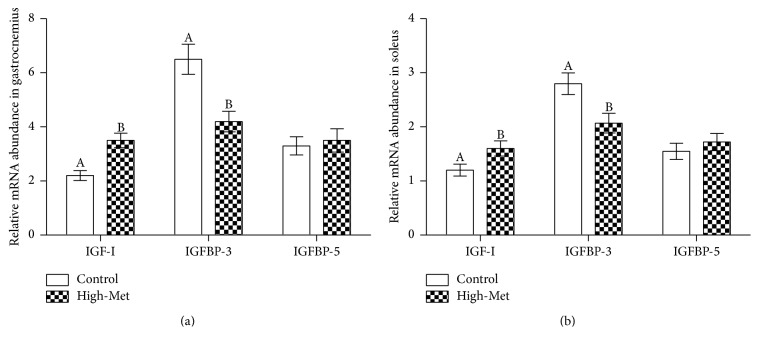
Results of high-Met diet upon the level of mRNA in gastrocnemius (a) and soleus (b). Data is presented as means ± SEM (*n* = 8).  ^A,B^The varying characters indicate the statistically significant differences between the high-Met and control groups (*P* < 0.05).

It appears that a high-Met diet leads to surges in the concentration of IGF-I found within the high-Met samples in comparison to the control group (Figure 2). As can be observed in Figure 3(a), the high-Met diet also led to decreased atrogin-1 and MuRF1 mRNA, observed in the gastrocnemius, with values below that of the control group (*P* < 0.05). At the same time, Figure 3(b) demonstrates that a high-Met diet did not affect atrogin-1 or MuRF1 mRNA levels within soleus contexts in comparison to the base group (*P* < 0.05).  Figure 4(a) demonstrates that a high-Met diet had no effect in the gastrocnemius on IGFBP-5 mRNA compared to the control group. Conversely, IGF-I mRNA levels were elevated significantly (*P* < 0.05), and IGFBP-3 mRNA significantly declined (*P* < 0.05) for the gastrocnemius as compared to the control group. Identical trends were observed in the soleus, with high-Met diet raising IGF-I mRNA levels (*P* < 0.05) and dropping IGFBP-3 mRNA (*P* < 0.05), with no statistically significant effect on IGFBP-5 mRNA levels (Figure 4(b)).

## 4. Discussion

The current research suggests that feeding arthritic rats a high-Met diet causes their food consumption to increase, as well as overall weight, in addition to serum IGF-I levels. Plasma IGF-I response levels to dietary Met have been noted in existing research [14]. However, as discussed by Nagao and colleagues [15], the regulatory implications of dietary Met were autonomous from plasma IGF-I concentration variations. High-Met diets resulted in raised serum level of IGF-I, which may be responsible for the arthritic intensity in rats. In the case of healthy humans with typically functioning IGF-I, IGF-I will lead to greater muscle protein synthesis [16]. Also, IGF-I has the capability to minimize the effects of negative nitrogen balancing throughout caloric reductions and an increased muscle mass within hypophysectomized animals [6]. Studies have also discussed the potential for IGF-I administration to minimize the debilitating effects of chronic arthritis [13]; such conclusions may accommodate to the present findings that a high-Met diet may moderately inhibit arthritic hindrances relative to body weight and overall reduce debilitating aspects of arthritis.

Arthritis behaves differently between the gastrocnemius and soleus, whereby muscle atrophy is observed to be much greater in the former than in the latter. Upregulation of arthritis-induced atrogenes atrogin-1 and MuRF1 showed values for the gastrocnemius to be higher than in samples extrapolated from the soleus. Furthermore, arthritic implications on IGF-I and IGFBP-3, in addition to factors of myogenic maintenance, are greater in the gastrocnemius. Preserving oxidative as a means to prevent muscle atrophy over several chronic diseases is evidenced by the oxidative muscle displaying efficacious responses to the antioxidant system relative to the glycolytic muscle [17]. Reactions from IGF-I administration follow the same pattern. In the arthritic rats' gastrocnemius, high-Met diets lowered atrogin-1 and MuRF1. Conversely, a high-Met diet had no effect on atrogin-1 and MuRF1 gene expression in the arthritic rats' soleus samples.

IGF-I regulation and relative proteins which bind to it behave in different ways in the liver and skeletal muscle. In the present research, it was found that high-Met diets facilitated the circulation of IGF-I; however, no effect was observed in skeletal muscle IGF-I mRNA. In the same vein, IGF-I generation in the liver varies as opposed to its mechanism in all other organs and tissues. Administration of clenbuterol, for instance, a *β*2-adrenoceptor agonist, catalyzes a rise in IGF-I and IGFBP-5 gene expression as observed soleus contexts, but circulation of IGF-I is inhibited [18].

Some researches purported that locally procured IGF-I should be prioritized over IFG-I as a preserver of muscle strength and mass [6]. Along these lines, there is elevated IGF-I gene expression found within gastrocnemius contexts only, within arthritic rats. Yet muscle deterioration is more apparent in the former than the latter [13]. A fractional amendment to IGF-I circulation was found to have a great efficacy in catalyzing weight gain as well as gastrocnemius mass within the subject pool of arthritic rats.

The results of this study are indicative of a high-Met diet providing a crucial contribution to inhibiting muscle atrophy. Surges in the level of IGFBP-3 were significantly evident in soleus samples of the arthritic rats, however, not as steep as those noted from the gastrocnemius [1]. Increases in IGFBP-3 are also evident in existing literature; after 48-hour period following injury, this time is denoted as the preliminary recovery stage, during which inflammatory cells in the muscles are abundant, particularly in the vicinity of macrophages [19]. The current study ascertained that IGFBP-3 is primarily abundant in macrophages in the near vicinity of the afflicted tissue [19]. Overexpressing IGFBP-3 and inhibiting the binding of IGF-I receptors have autonomous IGF-I effects in reducing the overgeneration of cells [20]. In addition, IGFBPs prevention utilizing IGF-I aptamer gave rise to augmented tissue regeneration and benefitted from increases in recovery duration, from fast twitch as a result of myotoxic damage [5]. It may be deduced that reductions in IGFBP-3 in muscles following high-Met diets can indeed supplement the anabolic effect of IGF-I.

To conclude, data procured in this study strongly support the hypotheses and existing data that attenuation of arthritis may be obtained through an enhanced high-Met diet. In the case of the lab rats, high-Met diets catalyzed a surge in serum IGF-I levels and inhibited the level of a high-Met diet for atrogin-1, as well as MuRF1. Decreases in IGFBP-3, as well as the active engagement in myogenic regulatory elements, have been shown to have a positive effect in muscle retention.
